# When Parallel Roads Meet: Orchestrating Collaborations Between Regulatory, Ethical, and Business Partners in Translational Medicine

**DOI:** 10.3389/fmed.2019.00087

**Published:** 2019-05-03

**Authors:** Uri Tabori, Joseph Ferenbok, Emmanuel Thomas, Joost Frans Swart Thomas, Salvatore Albani, Vicki Seyfert-Margolis, Emilie Sauvage

**Affiliations:** ^1^Department of Paediatrics, Hospital for Sick Children, Toronto, ON, Canada; ^2^Institute of Medical Sciences, University of Toronto, Toronto, ON, Canada; ^3^Leonard M. Miller School of Medicine, University of Miami, Miami, FL, United States; ^4^Department of Pediatric Rheumatology & Immunology, University Medical Center Utrecht, Utrecht, Netherlands; ^5^Translational Immunology and Inflammation Centre, SingHealth, Singapore, Singapore; ^6^MyOwnMed, Inc., Bethesda, MD, United States; ^7^Institute of Cardiovascular Science, University College London, London, United Kingdom

**Keywords:** translational medicine, regulators, innovators, business, patients

The exponential growth in technological abilities and biological understanding of diseases result in the advancement of novel interventions and therapeutics which may dramatically impact patients. Despite this significant progress in biomedical science and technology, the efficiency of clinical product development (i.e., drugs, medical devices, and medical procedures) is not improving and may actually be decreasing. Innovations arising from medical research are nowadays facing important hurdles that prevent their timely implementation into patient management, treatment and health policies (1).

Although promotion of standards, recording and reporting information is a key stepping stone to an effective and safe healthcare system, the high volume of bureaucracy hinders an early introduction into the market (1–3). At the same time, the costs of drug development continue to rise.

We require a team of stakeholders to translate the overwhelming rate of inventions into clinical care (Figure 1). The five components of such a team are (1) The innovators, most commonly the Academia, (2) Industry, key to fund and facilitate the innovation, (3) The research ethics board, mostly located in clinical institutions and responsible for the safety of patients and society and (4) The national/regional regulatory agencies which take into account ethical, feasibility and financial aspects important for the ultimate implementation. The last (5) and perhaps the most important stakeholder are the patients and their representatives.

**Figure 1:**
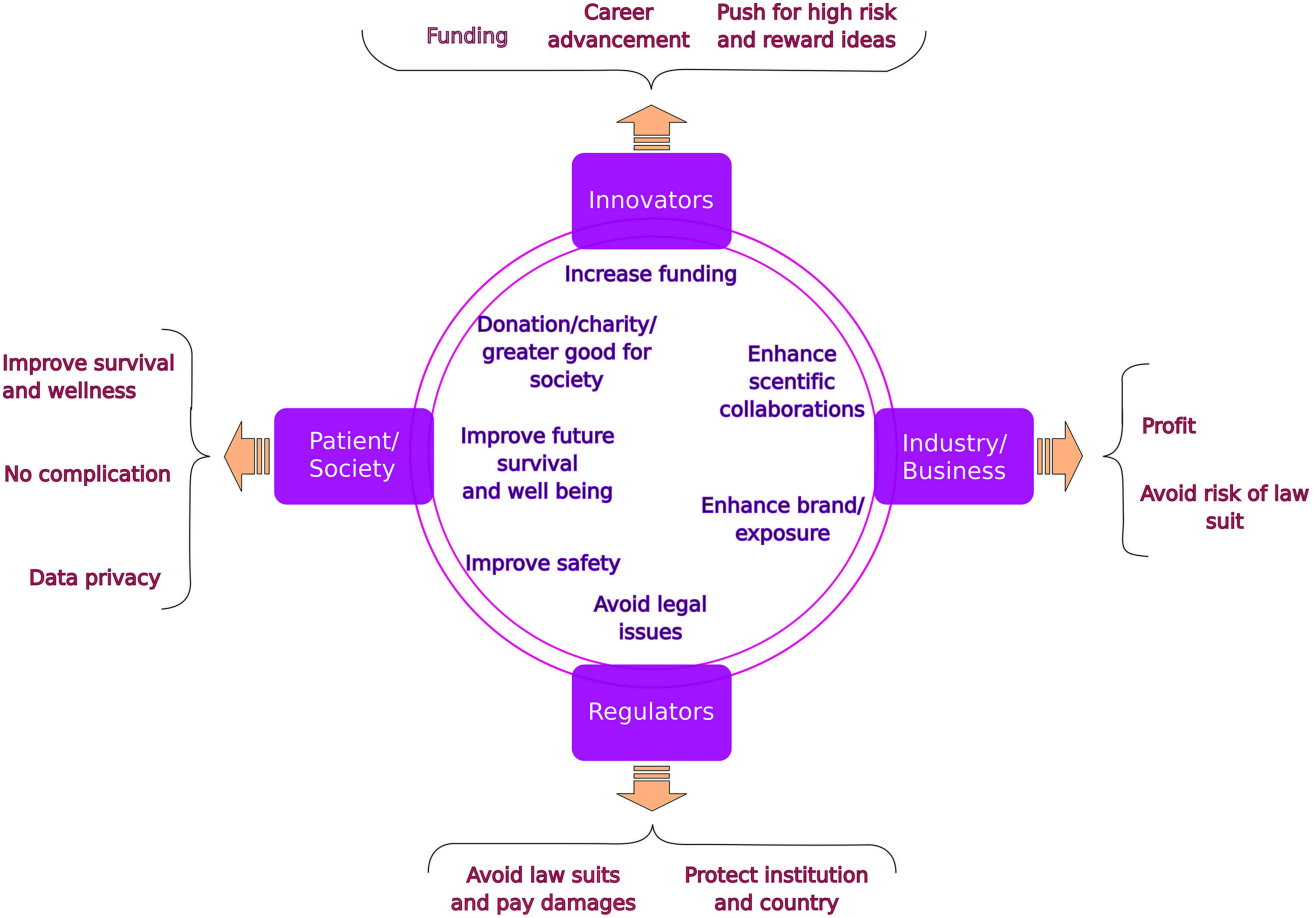
Stakeholders in translational medicine: common goals (in the circles) and specific concerns (in the braces).

This paper is a part of a Frontiers research topic entitled “The Silent Cry: How to Turn Translational Medicine Toward Patients and Unmet Medical Needs” which is a larger series collecting other position papers addressing issues related to early discovery, patenting, animal work, preclinical and other aspects of clinical trials. Coming from the observation that translational process is highly inefficient in the current healthcare state, this series was created to raise awareness on the different hurdles of the process. The list of all topics developed in the *silent cry* series can be found on the Frontiers webpage: (4). Nonetheless, the focus point of this article is the relationships between the academic, the business, the ethical and the regulatory partners.

Together, the tension between potential benefits from innovation and the fallout upon mistakes in safety, legal repercussions or financial loss results in a rough path of progress and frustrations for all sides in this endeavor. For each of the partners there are specific benefits but most importantly risks to be aware of. The unique risks for each partner result in delays and are considered bureaucratic hurdles in the translational medicine path. Only by understanding the risks and benefits for each partner, a project can be successful in a timely manner (5, 6).

In the present paper we lay out the individual concerns of each partner as well as the common beneficial components (Figure 1), which will potentially help improving the interactions and easing the path to translate innovations into clinical practice. Other major hurdles which exist in this path from early discovery to clinical trial and ultimately implementation are discussed in related articles to the *silent cry* series.

The initial stakeholders are the researchers who cherish the invention as their own “child” and strive to get the discovery published in a high impact journal while applying for funding. In their view, the potential benefit to patients makes it “unethical” not to proceed with patient interaction early on. It is common that they overlook important steps such as patent, appropriate financing, necessary technical and regulatory steps and ultimately the distribution of the innovation to the public. This can affect the maturation of their “child”.

Each business partner, on the other hand, needs to observe the invention through the lens of financial gain. Protection of any invention by intellectual property is necessary but by itself not sufficient for industry to invest in the product. Unidentified risks and lack of clear benefits can discourage the Industry partner from joining this endeavor. Industry usually wants to see a clear horizon of patient-numbers, benefits, safety and economic value (e.g., will a drug warrant reimbursement by payers and will it be beneficial enough to sell on the market). Therefore, innovations in small populations such as most childhood diseases are a major hurdle. Potentially small volumes of cases, possible legal ramifications and reputational damage of adverse events in children, competition with larger (adult) indication groups result in tensions between the innovator and the business partner.

Although the mandate of the Instutional Review Board (IRB) is to advance research in a safe and ethical way toward patient and society, the institutional protection and avoidance of potential individual law suits commonly interfere with this initial mission. The more innovative and invasive the discovery, the higher is the risk for the IRB. This tension creates automatically a higher need for safety measures resulting in the much discussed bureaucratic hurdles observed by the innovators. Furthermore, if potential financial profit is involved, further tension raises between the IRB and the industry partner.

Last but not least are the regulatory agencies responsible for protecting the population from adverse events while also considering both clinical and financial implications. Importantly, the extensive testing for new therapeutics or medical devices often represents large and risky financial commitments from manufacturers (7). Only well established companies are able to absorb these level of costs, which prevents new players from entering the market competition. The risks without yet clear long term benefit to the patients and society result in risk aversion even more so when approvals require additional steps, time and costs in high-risk products. The tension between the innovator/industry and the regulatory agencies is also increased when the decision process is not transparent and/or the individuals responsible for these decisions cannot be contacted.

In order to improve the integration between the abovementioned four partners in this part of translational medicine, it is important for all stakeholders to first appreciate each partner's roles and priorities, and to guarantee their independency and role in the process. To achieve efficient project progression it is essential to increase risk tolerance and emphasize the potential short and long term benefits to each partner. Specific tools to achieve these goals include a “concierge service” which means early on involvement of industry partnership and research collaboration experts. These experts, speaking the same industry jargon, will know how and when to approach the right business partners. They can also be involved in the regulatory issues further in the process. One should not hold back on approaching high up individuals in the involved companies to ensure sustained partnership.

Many countries are moving into regional and nationwide IRB agencies which will reduce diversity and increase transparency in decision making and shorten times for large projects. Involvement of innovators in the IRB committees and continuous learning and discussions with IRB chairs will also facilitate trust and reduce risk aversion. When approaching the regulatory agencies, seeking experts and meeting the individuals responsible for specific applications will result in better and faster outcomes. The use of external experts and advocates might also support this goal.

Finally, patient advocates (8, 9) are extremely important from early on as they also play key role in the discussions with IRB and regulatory agencies. They can guide the innovator and industry in the priorities of the end users of the product. Specific care should be spent on the role of smart (dynamic) consents covering the current issues as well as the future potential use of patient data and tissues. One should explore the potential collaborations with industry and non-academic stakeholders.

When balancing technological innovations, new medical concepts and deeper understanding of human biology translational projects can transform disease management and thereby improve patient outcome. Ethics, health and economics are all at stake and therefore a careful approach including participation of all stakeholders is required. Understanding the risk and benefits for each partner in this journey and keeping active representation of each of the partners in every decision making step will reduce the tension and is the most fruitful way forward.

## Author Contributions

ES, UT, and JF wrote the manuscript with support from ET, JS, and VS-M. SA helped supervise the project.

### Conflict of Interest Statement

UT receives funding from AACR/SU2C BMS supported catalyst grant. VS-M is doing business with Conopco Inc (d/b/a Unilever), Janssen Research and Development LLC, Kintai Therapeutics Inc, Novartis Pharma AG, Otsuka America Pharmaceutical Inc, Sanofi US. The remaining authors declare that the research was conducted in the absence of any commercial or financial relationships that could be construed as a potential conflict of interest.
